# Nanomedicines Meet Disordered Proteins: A Shift from Traditional Materials and Concepts to Innovative Polymers

**DOI:** 10.3390/jpm12101662

**Published:** 2022-10-06

**Authors:** Bruno Rizzuti

**Affiliations:** 1 CNR-NANOTEC, Sede Secondaria Rende (CS), Department of Physics, University of Calabria, 87036 Rende, Italy; bruno.rizzuti@cnr.it; 2 Institute of Biocomputation and Physics of Complex Systems—Joint Unit GBsC-CSIC-BIFI, University of Zaragoza, 50018 Zaragoza, Spain

**Keywords:** nanomedicines, copolymers, intrinsically disordered proteins, HPMA, EPR, nanocarriers

## Abstract

Water-soluble nanomedicines have been widely studied for the targeted delivery of drugs for a very long time. As a notable example, biomaterials based on *N*-(2-hydroxypropyl) methacrylamide (HPMA) copolymers have been under investigation for nearly half a century. In particular, anticancer drug carriers have been developed under the assumption that the leading mechanism with a therapeutic impact on solid tumors is the enhanced permeability and retention (EPR) effect, which dates back more than three decades. Nevertheless, these (and other) materials and concepts have encountered several barriers in their successful translation into clinical practice, and future nanomedicines need improvements in both passive and active targeting to their site of action. Notions borrowed from recent studies on intrinsically disordered proteins (IDPs) seem promising for enhancing the self-assembly, stimuli-responsiveness, and recognition properties of protein/peptide-based copolymers. Accordingly, IDP-based nanomedicines are ready to give new impetus to more traditional research in this field.

Polymer nanomedicines are water-soluble macromolecules that release bioactive molecules for therapeutic effect. They are rationally designed to have maximal solubility, stability, and pharmacokinetic properties, and are expected to reach their target cells with high accuracy and minimal impact on the surrounding healthy tissues [1]. Nanocarriers are especially considered for chemotherapy, with other important applications including anti-inflammatory and anti-microbial medications, as well as the treatment of virtually any disease that may benefit from the tailored delivery of drugs. In spite of their long history, huge recent advances, and bright potential in the development of polymer nanomedicines, in many cases, there are still some issues hampering their use in the clinical practice [2]. To overcome these obstacles, long-standing materials and concepts have been updated with new findings in other areas of polymer chemistry and physics. Intrinsically disordered proteins (IDPs) are heteropolymers with unusual properties and highly specific tunability features [3], and they may help us to improve the design of drug delivery systems in the near future.

To illustrate these points, a clear example is the synthetic polymer *N*-(2-hydroxypropyl) methacrylamide (HPMA) [4]. Since the 1970s, this polymer has been combined with proteins and oligopeptide sequences to obtain nanocarriers for the delivery of bioactive compounds [5]. HPMA was originally developed as a substitute of blood plasma for transfusions; as a consequence, after some preliminary studies on various cell lines to exclude toxicity, it was extensively tested in vivo on several animal models [6]. Its administration in saline solution showed no pyrogenic effect, and subcutaneous implants did not provoke an immune response or lead to the production of detectable antibodies. Therefore, it was concluded that the HPMA polymer could be considered highly biocompatible and a strong drug nanocarrier candidate with reasonably positive potential for use in human clinical trials.

In a recent landmark literature review in the Journal of Personalized Medicine, Chytil and coworkers [7] summarized the current status and perspectives on the use of HPMA copolymer-based nanomedicines in controlled drug delivery, starting with the safety features of the parent homopolymer. HPMA copolymers and drug conjugate structures with narrow dispersity can be obtained using modern synthesis techniques. More complex structures have also been produced, including diblock, multiblock, grafted, and star geometries [8]. Self-organized supramolecular assemblages, such as micelles, can also be built to obtain long-circulating nanocarriers with various degrees of biodegradability [7,9]. Nevertheless, in spite of the great wealth of research carried out so far, no prodrug based on HPMA has yet passed clinical-phase trials on patients and reached the market. Although HPMA copolymers still remain among the most prominent candidates for nanomedicines, the current situation calls for improving their formulation in the near future.

The development of nanomedicines targeting solid tumors, including HPMA-based nanocarriers, is often discussed in connection with the enhanced permeability and retention (EPR) effect [10]—a well-known phenomenon that was first discovered in the 1980s and, therefore, has a long story that spans more than three decades. Cancer is a major threat to human health in most developed countries, and therapies consisting of the delivery of cytotoxic drugs lead to unwanted side effects due to poor selectivity towards the diseased cells. However, soluble macromolecular drugs with anticancer properties tend to benefit from the EPR effect, which is based on their propensity to accumulate in tumor tissues due to increased permeability of the local vessels, a high degree of vascularization, and deficiencies in lymphatic drainage of the interstitial region. A Special Issue on EPR effect-based targeted nanomedicine [11] was recently published in the Journal of Personalized Medicine and it includes, among other works, a detailed historical perspective [12], discussion of the significance of the EPR concept [13], and recent advances in its importance for cancer treatment [14].

Nevertheless, as explicitly pointed out by Chytil and coworkers in the Special Issue cited above [7], a number of key paradigms in cancer nanomedicine have recently been challenged, including the EPR effect. Among other reasons, it has been recently reported [15] that the majority of nanoparticles enter tumors through endothelial cells by means of an active process, rather than through gaps among endothelial cells in the tumor blood vessels. Along with other criticisms towards the centrality of the EPR effect [16], these new findings encourage alternative strategies based on exploiting active pathways to achieve the accumulation of nanomedicines in tumor tissues. So, the big question is: where do we move next in the formulation of copolymers for therapeutic purposes to improve both passive and active targeting?

Answering such question represents one of the biggest challenges in this field and requires careful consideration of the latest novelties in other closely related fields. The example of HPMA clearly shows that, in spite of the underwhelming results obtained so far with regard to translation into clinical practice, there is nothing fundamentally wrong with the use of such a polymer, and abandoning it would obviously not be a wise strategy. In contrast, it is natural to move our attention towards improving the combination of other nanomaterials in the formulation of more suitable copolymers [9]. In this respect, the EPR effect can still provide important guidelines on some significant features that such materials should possess. For instance, it is known that retention and accumulation effects in a solid tumor depend not only on the molecular weight (MW) of the macromolecular species but also on other key physical properties—more prominently, on their charge and hydrophilic/hydrophobic character [17,18]. Thus, in the case of protein or peptide copolymers, the proteinaceous component could help to modulate such characteristics.

One of the most recent trends in the biotechnology field is the increasing focus on natively unfolded proteins/peptides (i.e., IDPs), as well as intrinsically disordered regions (IDRs) found within well-folded proteins. One of the reasons is their great structural and dynamic versatility [19], which is strictly related to their conformational flexibility. Another reason is that IDPs/IDRs are significantly more abundant in eukaryotic than prokaryotic cells [20], suggesting that more complex functionalities benefit from an increase in the degree of disorder of the macromolecules involved in such tasks. Remarkably, charge and hydrophobicity (key factors in modulating passive targeting that relies on the EPR effect, as mentioned above) are the two most important features that identify IDPs [21], dictate their properties, and give them some unique adaptability features. In their interactions with different substrates, IDPs can have a surprisingly diverse range of behaviors [22]: folding-upon-binding, polymorphic collapse into alternative structures, context-dependent conditional folding, or the formation of fuzzy (but not entirely aspecific) heterogeneous contacts. These properties are important for molecular recognition, which, in turn, is a fundamental aspect of active targeting in nanomedicines.

In light of these features, in addition to being increasingly considered on their own as druggable targets in many pathologies [23,24], IDPs are also promising nanomaterials that can be combined in nanocarriers for medical and biotech applications. For instance, IDP amphiphiles are diblock polymers that contain a hydrophilic IDP domain attached to a hydrophobic peptide sequence [25] or dendritic tail [26]. These molecules self-assemble into micelle nanocarriers for the encapsulation of chemotherapeutics or other drugs [27], and they may exhibit a pH-induced phase transition from a low-dispersity spherical shape to an elongated worm-like one [26], representing a potential mechanism for the hold-and-release of cargoes. In fact, IDP-based constructs have unique properties of responsiveness towards pH, temperature, and molecular crowding conditions [28], which are key factors in the microenvironment of diseased tissues. IDP regions can also form nanocages [29] or be attached to the surface of conjugated nanoparticles [30], therefore constituting a stealth layer to modulate their circulation half-life. In the former case, a tumor-targeting affibody peptide could also be fused to the nanocage to obtain a functional theragnostic platform [29].

In summary, new materials and properties (such as those stemming from current studies on IDPs) are currently being investigated to advance the formulation and controlled delivery of nanomedicines. Acknowledged materials (including HPMA-based copolymers) and phenomena (such as the EPR effect), with a history spanning several decades, are expected to benefit such novel ideas in overcoming the difficulties faced on the road towards their clinical use in patients. This will further enrich the arena of actively targeted protein-based copolymers, which already encompass various targeting ligands (e.g., monoclonal antibodies, immunoglobulins, lectin-like domains, affibody molecules, tailored peptides, etc.). The cross-pollination of concepts from different fields will help us improve our capacity to suggest innovative approaches and unlock updated strategies to target tumors and other diseases.

## Data Availability

Not applicable.
